# The biased apelin receptor agonist, MM07, reverses Sugen/hypoxia-induced pulmonary arterial hypertension as effectively as the endothelin antagonist macitentan

**DOI:** 10.3389/fphar.2024.1369489

**Published:** 2024-04-09

**Authors:** Thomas L. Williams, Duuamene Nyimanu, Rhoda E. Kuc, Richard Foster, Robert C. Glen, Janet J. Maguire, Anthony P. Davenport

**Affiliations:** ^1^ Experimental Medicine and Immunotherapeutics, University of Cambridge, Addenbrooke’s Hospital, Cambridge, United Kingdom; ^2^ School of Chemistry, Astbury Centre for Structural Biology, University of Leeds, Leeds, United Kingdom; ^3^ Department of Chemistry, Centre for Molecular Informatics, University of Cambridge, Cambridge, United Kingdom; ^4^ Department of Surgery and Cancer, Biomolecular Medicine, Imperial College London, London, United Kingdom

**Keywords:** GPCR, apelin receptor, apelin, MM07, biased signalling, pulmonary hypertension, animal model, cardiovascular

## Abstract

**Introduction:** Pulmonary arterial hypertension (PAH) is characterised by endothelial dysfunction and pathological vascular remodelling, resulting in the occlusion of pulmonary arteries and arterioles, right ventricular hypertrophy, and eventually fatal heart failure. Targeting the apelin receptor with the novel, G protein-biased peptide agonist, MM07, is hypothesised to reverse the developed symptoms of elevated right ventricular systolic pressure and right ventricular hypertrophy. Here, the effects of MM07 were compared with the clinical standard-of-care endothelin receptor antagonist macitentan.

**Methods:** Male Sprague-Dawley rats were randomised and treated with either normoxia/saline, or Sugen/hypoxia (SuHx) to induce an established model of PAH, before subsequent treatment with either saline, macitentan (30 mg/kg), or MM07 (10 mg/kg). Rats were then anaesthetised and catheterised for haemodynamic measurements, and tissues collected for histopathological assessment.

**Results:** The SuHx/saline group presented with significant increases in right ventricular hypertrophy, right ventricular systolic pressure, and muscularization of pulmonary arteries compared to normoxic/saline controls. Critically, MM07 was as at least as effective as macitentan in significantly reversing detrimental structural and haemodynamic changes after 4 weeks of treatment.

**Discussion:** These results support the development of G protein-biased apelin receptor agonists with improved pharmacokinetic profiles for use in human disease.

## 1 Introduction

Pulmonary arterial hypertension (PAH) is a progressive disease characterised by functional and structural changes of the pulmonary vasculature, resulting in increased pulmonary vascular resistance, and fatal right-sided heart failure (Thenappan et al., 2018; Ruopp and Cockrill, 2022). An imbalance of vasoconstrictors and vasodilators contributes to increased proliferation of pulmonary arterial endothelial cells (PAECs), pulmonary arterial smooth muscle cells (PASMCs), and fibroblasts, resulting in remodelling of the vessel wall and progressive occlusion of pulmonary arteries (Morrell et al., 2009; Alastalo et al., 2011; Schermuly et al., 2011). PAH can be hereditary, idiopathic, or triggered by drugs/toxins, hypoxia, or inflammation (Thenappan et al., 2018). Mutations in the bone morphogenetic protein type 2 receptor (BMPR2), and associated proteins in the transforming growth factor-β pathway, underlie most heritable forms of PAH (Graf et al., 2018; Happé et al., 2020).

Current treatment options are aimed at targeting three key signalling pathways: (1) the prostanoid IP receptor, using agonists such as prostacyclin, epoprostenol, selexipag; (2) nitric oxide, via activation of soluble guanylate cyclase (sGC) with stimulators such as riociguat, or phosphodiesterase 5 inhibitors (PDE5), for example, sildenafil and tadalafil; and (3) endothelin receptors, using antagonists including the mixed ET_A_/ET_B_ antagonists bosentan and macitentan, or the ET_A_ selective antagonist ambrisenstan (Abraham et al., 2023). Additionally, sotatercept, a first-in-class inhibitory fusion protein consisting of the Fc domain of human IgG linked to the extracellular domain of human activin type II receptor (ActRIIA), which acts as a ligand trap for selected transforming growth factor-β superfamily members, improved exercise capacity in PAH patients in a recent phase 3 clinical trial (Hoeper et al., 2023). Sotatercept is on track for Food and Drug Administration (FDA) approval for the treatment of PAH in early 2024 (Torbic and Tonelli, 2024).

Despite the range of treatment options, current therapeutics are typically limited to targeting pulmonary vasoconstriction and remodelling in PAH, having little to no beneficial action directly on the failing heart, and consequently mortality rate remains high (3-year mortality of ∼21%) (Chang et al., 2021). The identification of novel therapeutics that provide beneficial cardiac effects will be crucial to improve PAH patient outcome.

Apelin is an endogenous peptide ligand that targets the class A G protein-coupled apelin receptor (O’Dowd et al., 1993; Tatemoto et al., 1998). Both apelin and the apelin receptor are widely expressed in various tissues and cell types, including endothelial cells, smooth muscle cells and cardiomyocytes, where they mediate vasodilatation and positive cardiac inotropy in health and disease (Kleinz et al., 2005; Maguire et al., 2009; Marsault et al., 2019; Read et al., 2019; de Oliveira et al., 2022). Loss of BMPR2 in PAH is associated with an upregulation of the miR-130/301 miRNA family (Bertero et al., 2014; 2018), causing a subsequent reduction in the transcriptional activity of peroxisome proliferator-activated receptor gamma (PPARγ), and reduced expression of apelin (Alastalo et al., 2011; Yang et al., 2015). Studies have confirmed that apelin peptide levels are downregulated in both plasma (Goetze et al., 2006; Chandra et al., 2011) and in the pulmonary vasculature (Alastalo et al., 2011; Kim et al., 2013) of PAH patients compared to controls. Importantly, apelin receptor expression is preserved and may therefore be a potential therapeutic target.

Studies in animal models of PAH have demonstrated that infusion of apelin receptor agonist peptides is beneficial in attenuating features of PAH, reducing vascular muscularization, right ventricular systolic pressure (RVSP), and hypertrophy (Falcao-Pires et al., 2009; Alastalo et al., 2011; Yang et al., 2017; 2019). Further, in a recent clinical study of apelin in PAH patients, administration of apelin provided significant additive benefit, when given together with PDE5 inhibitor therapy, decreasing pulmonary vascular resistance, and increasing cardiac output and stroke volume (Brash et al., 2018). The data provide proof-of-principle that apelin receptor agonists could provide a novel strategy for treating patients with PAH, particularly if used in combination with current standard-of-care drugs.

However, as expected following activation of GPCRs, apelin peptides can stimulate both the G protein-dependent signalling pathways, and the β-arrestin pathway, which results in receptor internalisation, desensitisation, and, ultimately, loss of efficacy (Evans et al., 2001; Zhou et al., 2003; Masri et al., 2006). G protein-dependent signalling at the apelin receptor is cardioprotective however, genetic and pharmacological studies suggest that β-arrestin recruitment and signalling induces detrimental effects, including increased cardiac hypertrophy and fibrosis (Scimia et al., 2012). Functionally selecting for activation of the G protein pathway, without recruiting β-arrestin to the apelin receptor, may further enhance the therapeutic benefit in cardiovascular diseases such as PAH.

We have previously reported the design and characterisation of a novel cyclic apelin mimetic, MM07, which showed significant functional selectivity for the G protein pathway at the apelin receptor (Brame et al., 2015). In human saphenous vein assays, MM07 showed comparable potency to native [Pyr^1^]apelin-13, and demonstrated ∼1300-fold selectivity for the G protein pathway. Experiments in rats and humans demonstrated that MM07 was more efficacious than [Pyr^1^]apelin-13 *in vivo*, in increasing cardiac output or forearm blood flow in healthy volunteers, with no evidence of receptor desensitisation (Brame et al., 2015).

The superior beneficial cardiovascular effects of MM07 prompted us to investigate whether this peptide would prevent PAH progression in a rat monocrotaline model of the disease. We observed significant reversal of established PAH, characterised by decreased right ventricle (RV) hypertrophy, pulmonary artery muscularisation and RVSP (Yang et al., 2019). The aim of the current study is to demonstrate that MM07 is also effective in slowing disease progression in the more severe Sugen/hypoxia (SuHx) rat model of PAH. This approach combines vascular endothelial growth factor (VEGF) receptor blockade using Sugen (SU-5416), with chronic hypoxia, to induce severe angio-proliferative pulmonary arterial hypertension, that is accompanied by intimal vascular wall thickening and the formation of lesions in the pulmonary vasculature that are indistinguishable from those observed in the human disease (Taraseviciene-Stewart et al., 2001; Abe et al., 2010; Nicolls et al., 2012; de Raaf et al., 2014).

**FIGURE 1:**
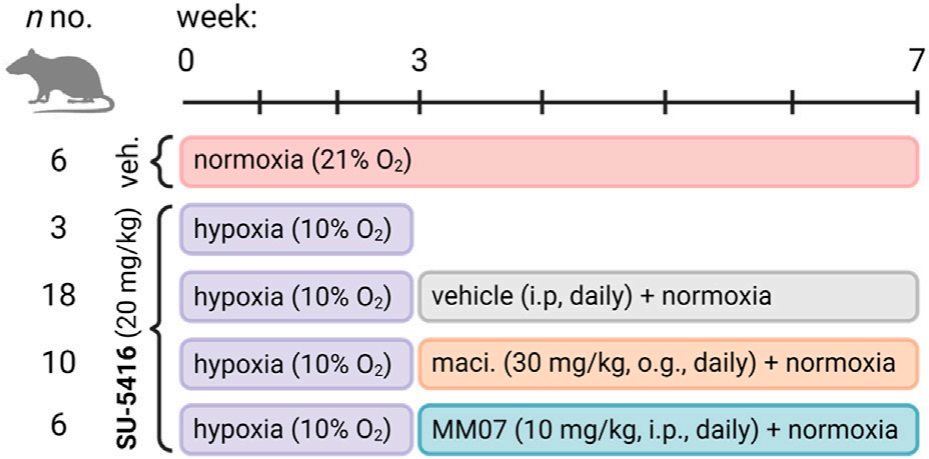
Experimental study design for the Sugen/hypoxia reversal study. Rats (*n* numbers for experimental groups provided) were administered once with vehicle (subcutaneously) or Sugen (SU-5416, 20 mg/kg, subcutaneously) and transferred to hypoxic chambers for 3 weeks before being returned to normoxia and treated daily with either vehicle (intraperitoneally), macitentan (maci., 30 mg/kg, oral gavage), or MM07 (10 mg/kg, intraperitoneally). Schematic created with BioRender.com.

An overview of the experimental protocol we used in this study is provided in Figure 1. We compared the effects of MM07 treatment with the standard-of-care ET_A_/ET_B_ antagonist, macitentan, which is well validated in the SuHx model, providing beneficial effects on vascular and right ventricle remodelling and function (Drozd et al., 2017; Nadeau et al., 2017; Mamazhakypov et al., 2020). As vasodilators, apelin agonists acts as functional antagonists of endothelin mediated vasoconstriction (Maguire et al., 2009), where endothelin peptide (ET-1) levels are known to be elevated in hypoxic states (Grimshaw, 2007) and in PAH patients (Giaid et al., 1993). Additionally, ET-1 is upregulated in cultured human lung blood microvascular endothelial cells (Star et al., 2014) and in murine lung lysates in response to Sugen (Hung et al., 2014), and, further, in cancer patients treated with VEGF inhibitors (Camarda et al., 2022). Endothelin signalling has also been shown to be critical for the formation of plexiform lesions in a mouse SuHx model (Hung et al., 2014). We therefore hypothesised that MM07, as a physiological antagonist of endothelin signalling, would be as effective as macitentan in reversing SuHx symptoms and disease progression.

In this study, MM07 was observed to reverse SuHx induced right ventricle hypertrophy and vascular remodelling, and reduced RVSP, to a similar or better extent than macitentan. Our data suggest that future therapeutic strategies utilising G protein-biased apelin receptor agonists such as MM07 might provide alternative treatment options for patients who do not respond to the standard-of-care drugs, and may also offer further cardiovascular benefits when used with current dual or triple therapy strategies.

## 2 Materials and methods

### 2.1 Materials

Sugen (SU-5416) was purchased from Tocris Bioscience (Cat. No.:3037; Bristol, United Kingdom); DMSO (Cat. No.: 276855), PEG 400 (Cat. No.: D2438), benzyl alcohol (Cat. No.: 402834), polysorbate 80 or Tween 80 (Cat. No.: 59924) and carboxymethylcellulose (CMC) sodium (Cat. No.: 3037) were all obtained from Sigma-Aldrich (Gillingham, United Kingdom); 0.9% sterile saline was purchased from AquaPharm (Cat. No.: 15E22BC).

### 2.2 Ethics

The study was carried out under the authority of the Procedures Project Licence (PPL PAE2D0A13).

### 2.3 Preparation of suspension for SU-5416

A stock solution of 0.5% carboxymethylcellulose sodium (CMC) was prepared in sterile saline. SU-5416 was weighed and benzyl alcohol (1%) added, followed by Tween 80 (2%), DMSO (5%) and PEG 400 (5%), respectively. The resulting slurry was vortex-mixed and sonicated for 1 h at room temperature until a fine, uniform suspension was formed and 87% of 0.5% CMC subsequently added to make up to the final volume.

### 2.4 SuHx model of PAH

Adult male Sprague Dawley rats (150–200 g) were purchased from Charles River United Kingdom and allowed to acclimate to the animal facilities for 7 days before the start of the protocol (Figure 1). Rats were randomly assigned to two groups, hypoxia or normoxia. Rats in the hypoxia group received a subcutaneous injection of 20 mg/kg SU-5416 on day 0 and were immediately transferred to the hypoxic chamber (10% oxygen). Rats in the normoxia group received a subcutaneous injection of vehicle and were housed under normoxic conditions (21% oxygen). After 3 weeks, rats in the hypoxia group were further divided into three receiving macitentan (30 mg/kg; oral gavage: n = 10), MM07 (10 mg/kg, i. p.: n = 6), or vehicle (5 mL/kg; n = 18) daily for an additional 4 weeks all under normoxic conditions. Similarly, the vehicle-treated animals in the normoxic group received daily injections of vehicle for the additional 4 weeks, resulting in a total of four treatment groups.

Animals were fed a standard rat chow with water available *ad libitum* and housed under a 12 h light-dark cycle (on 07.00, off 19.00) with fluorescent lights, in an air-conditioned room (or hypoxic chamber) so that the temperature (21°C ± 2°C) and relative humidity remained stable. Nesting and cages were autoclaved prior to use and each cage individually ventilated, except when in the hypoxic chamber. Animals were marked with a tail tattoo and housed in cages of two to five and their body weights measured and recorded at least three times a week. All rats were housed in the same room for the duration of their time on study, whether in or outside of the hypoxic chamber, to control for any background noise generated by the hypoxic chamber. The position of the cages was periodically rotated to ensure that each one received a similar airflow profile throughout the study.

### 2.5 Assessment of cardiopulmonary function by catheterisation

On the terminal day (week 7), rats were anaesthetised and assessed for cardiopulmonary function. Specifically, right and left ventricular pressures were measured by cardiac catheterisation under terminal isoflurane anaesthesia as previously described (Yang et al., 2019). Body weight (g), heart rate (BPM), heart weight (g), and the Fulton index where determined, where the Fulton index is described as RV weight (g)/(RV + septum weight) (g) to provide a surrogate marker of RV hypertrophy. Additionally, RVSP and the pulmonary vascular resistance index (PVRI) was determined, where PVRI is described as (mean pulmonary arterial pressure–end diastolic pressure) (mmHg)/cardiac index. Terminal blood was taken from the vena cava for preparation of EDTA plasma samples, after which animals were killed by exsanguination. The trachea was then cannulated, the right bronchus tied off and the left lung inflated with 0.8% agarose and transferred to 10% buffered formalin to fix the lung *in situ* for histology. Rats were assessed for right ventricle hypertrophy, by removing the heart, carefully dissecting the right ventricle free from the left ventricle plus septum, and subsequently weighing these to determine the Fulton index (RV weight/LV + Septum weight). The heart and other lung tissue were dissected and frozen for histophathological analysis.

### 2.6 Histopathological analysis of rat tissues

At least five sections (4 µm) from each rat lung were stained with haematoxylin and eosin (H&E) and α-smooth muscle actin (α-SMA) for quantitative histological evaluation as previously described (Long et al., 2015; Yang et al., 2019). Histological analyses were performed by an independent histopathologist consultant blinded to the treatment groups, and were scored to assess the following features: blood vessel oedema, blood vessel smooth muscle mass, loss of small pulmonary vessels, obliteration of the lumen of small pulmonary vessels, disorganisation of the capillary sheet, disorganisation of the bronchiolar epithelium, loss of alveolar sac regularity, hyperinflation and expanded alveolar sacs, extent of pleural hyperplasia/fibroplasia/focal inflammation, and assessment of leukocyte accumulation around the pulmonary arteries and in the lung parenchyma. The scoring system was based on a scale of 0–5 where 0, represented no significant pathology, 1, occasional single pathology; 2, multiple focal pathologies in a single lung zone; 3, multiple focal pathologies in several lung zones; 4, multi-focal or diffused pathologies in single lung; and 5, multi-focal or diffused pathologies in several lung zones. A Mertz grid (150 × 200 µm) was applied per section to confirm alveolar area regularity.

For assessment of muscularisation, α-SMA staining was performed in sections spanning the expected vessel thickness from 20 to 60 µm in diameter across the lung tissue. Wall remodelling was scored as none, partial, or full muscularisation of vessels; the number of α-SMA neointimal lesions were also recorded. Automated brightfield images (16-bit, 0.325 × 0.325 μm scaling per pixel) were obtained using a Slide Scanner AxioScan.Z1 (Carl Zeiss Microscopy GmbH, Gottingen, Germany) microscope with a Plan-Apochromat 20x/NA0.8 M27 objective lens connected to a Hamamatsu Orca Flash camera. Acquired images were visualised using Orbit Image Analysis Software (v3.65).

### 2.7 Data analysis

Data are expressed as mean ± SEM, and individual data points are shown. All statistical analysis was performed in GraphPad Prism (v6). For statistical analysis, data were tested for normality using the D’Agostino-Pearson omnibus K2 test. As appropriate, one-way ANOVA with Tukey’s correction for multiple comparisons was used to determine significance where significance was met when *p* < 0.05.

## 3 Results

### 3.1 MM07 reversed SuHx induced right ventricle hypertrophy

**FIGURE 2:**
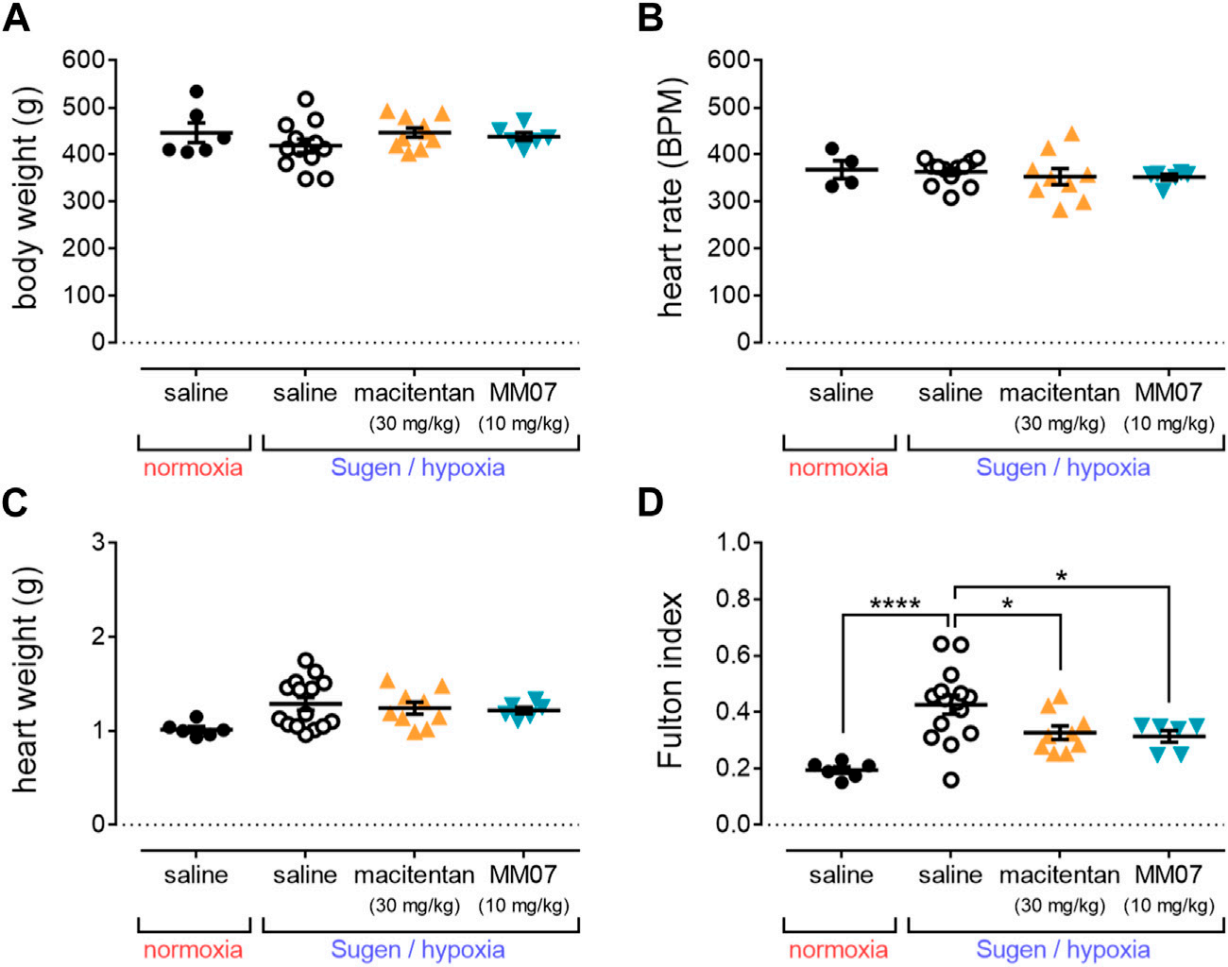
MM07 reduces right ventricular hypertrophy in SuHx treated rats. Graphs show: **(A)** body weight (g); **(B)** heart rate (beats per minute, BPM); **(C)** heart weight (g); or **(D)** Fulton index as an indicator of right ventricle hypertrophy, at the endpoint of the study. Treatment groups include normoxic saline, or Sugen/hypoxia followed by normoxia and saline, macitentan (30 mg/kg), or MM07 (10 mg/kg). Data expressed as individual data points, including mean ± SEM. Significance indicated by *p < 0.05, ****p < 0.00001.

At the end of the study (week 7), total body weights of rats (Figure 2A) assigned to control normoxia/saline, or SuHx in the presence of saline, macitentan (30 mg/kg), or MM07 (10 mg/kg) did not differ significantly. Additionally, heart rate (Figure 2B) was unchanged between treatment groups, demonstrating that neither macitentan or MM07 drug treatment had any notable effect on these physiological parameters.

Whilst total heart weights (g) (Figure 2C) of rats did not differ significantly, the Fulton index (Figure 2D) was significantly increased in rats treated with SuHx in the presence of saline versus normoxic/saline controls, consistent with the development of right ventricle hypertrophy in the SuHx model. Crucially, MM07 treatment significantly reversed the SuHx induced increase in the Fulton index to a similar extent as macitentan (Figure 2D), suggesting that the G protein-biased apelin ligand is effective in reducing right ventricle hypertrophy associated with PAH.

### 3.2 MM07 provided beneficial haemodynamic effects in SuHx induced PAH

**FIGURE 3:**
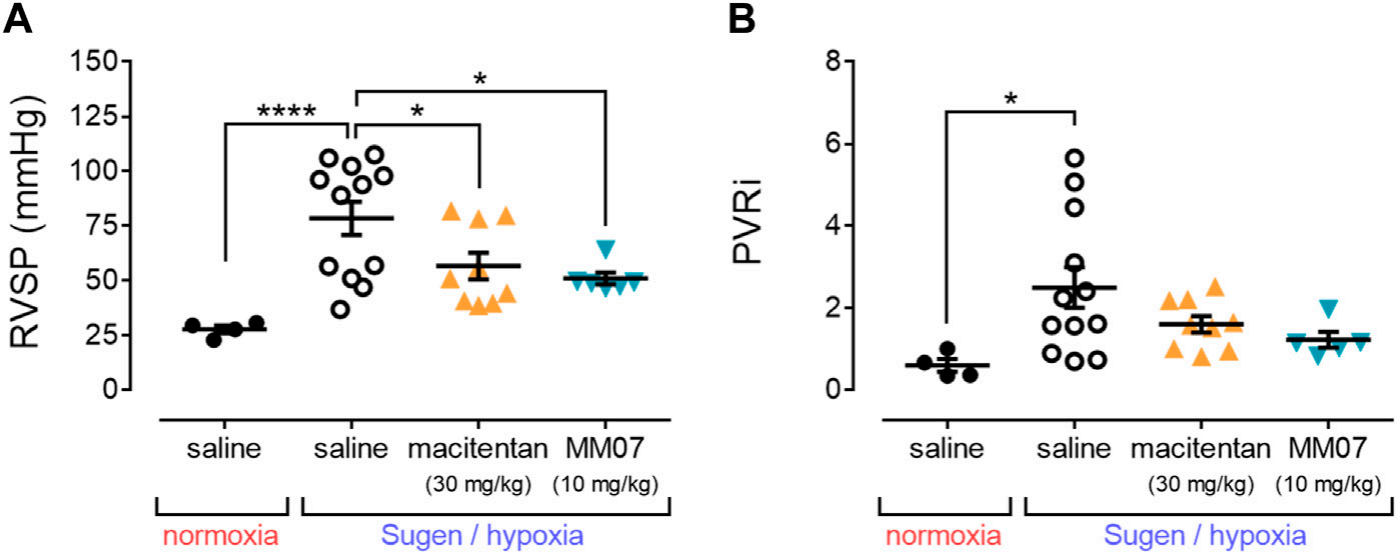
MM07 provides beneficial haemodynamic effects in SuHx treated rats. Graphs show: **(A)** right ventricular systolic pressure (RVSP, mmHg); or **(B)** the pulmonary vascular resistance index (PVRi), at the endpoint of the study. Treatment groups include normoxic saline, or Sugen/hypoxia followed by normoxia and saline, macitentan (30 mg/kg), or MM07 (10 mg/kg). Data expressed as individual data points, including mean ± SEM. Significance indicated by *p < 0.05, ***p < 0.0001.

SuHx treatment in the presence of saline resulted in a significant increase in right ventricular systolic pressure (RVSP) (Figure 3A) versus normoxic/saline controls, recapitulating a hallmark symptom of PAH. MM07 treatment significantly reversed raised RVSP in SuHx treated rats, as did the standard-of-care drug, macitentan (Figure 3A), suggesting both drug treatments resulted in an improved haemodynamic profile.

Further, the pulmonary vascular resistance index (PVRi) (Figure 3B) was calculated, and was shown to be significantly increased in SuHx treated rats in the presence of saline versus normoxic/saline controls. This increase was not significantly reversed in the presence of MM07 or macitentan, although there was a trend for both drug treatments to decrease PVRi.

### 3.3 MM07 reversed vascular remodelling in SuHx induced PAH

To further determine the beneficial effects of MM07 and macitentan on PAH pathogenesis, qualitative and quantitative assessment of the morphology and structure of pulmonary vessels was performed.

**FIGURE 4:**
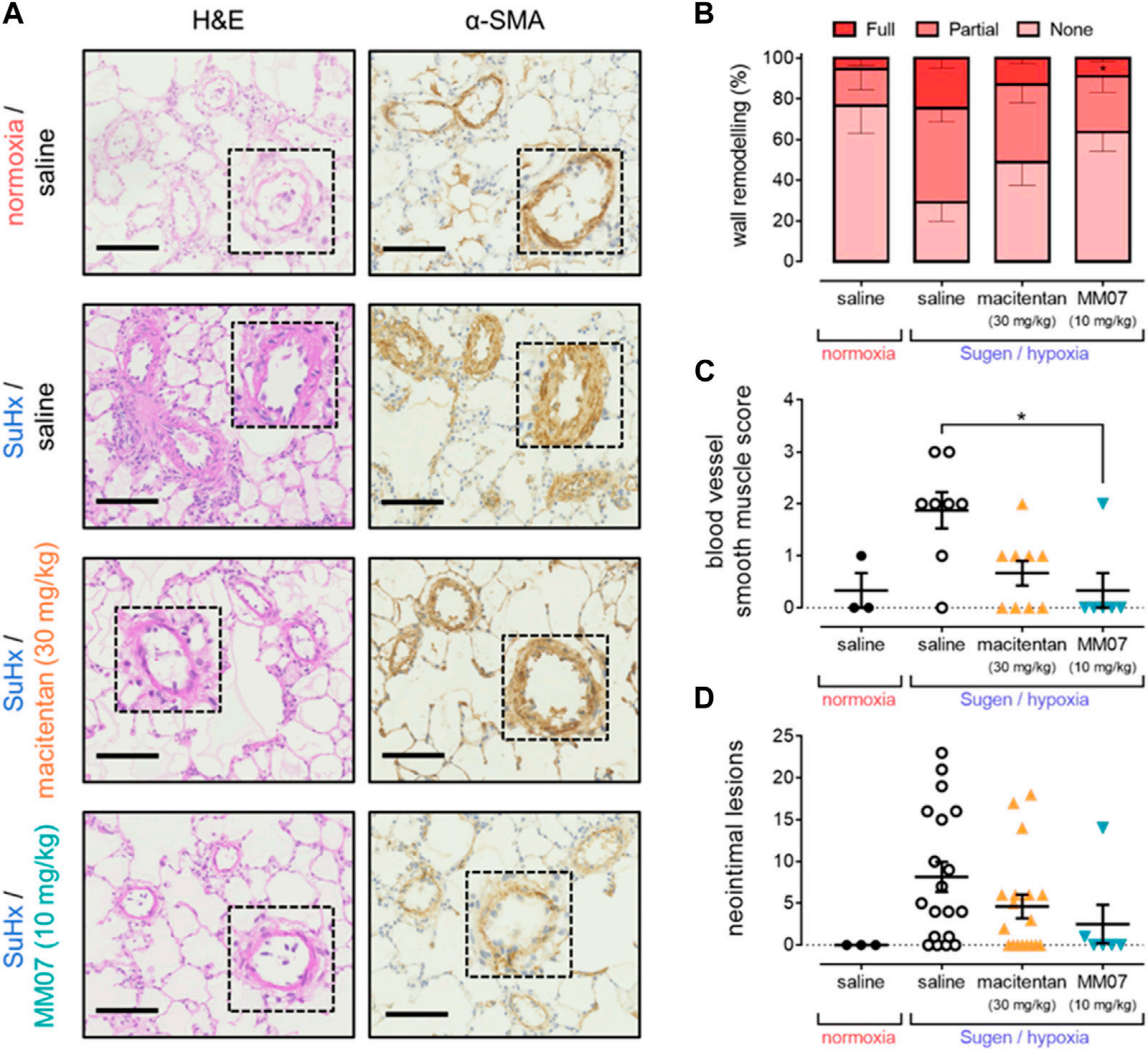
MM07 reverses SuHx-induced pulmonary vascular remodelling. **(A)** Visualisation of pulmonary artery vascular remodelling in representative sections of rat lung stained with haematoxylin and eosin (H&E; left) or α-smooth muscle actin (α-SMA; right). Images show tissues from normoxia/saline treated rats, or Sugen/hypoxia (SuHx) followed by normoxia and saline, macitentan (30 mg/kg), or MM07 (10 mg/kg). Dashed inserts show individual vessels magnified ×4. Scale bars indicate 100 μm. Graphs show: **(B)** percentages of fully, partially, or non-muscularised vessels in rat lung, representing vascular wall remodelling associated with PAH; **(C)** blood vessel smooth muscle score; or **(D)** number of neointimal lesions, quantified in sections of rat lung. Treatment groups include normoxic saline, or Sugen/hypoxia followed by normoxia and saline, macitentan (30 mg/kg), or MM07 (10 mg/kg). Data expressed as individual data points where applicable, including mean ± SEM. Significance indicated by *p < 0.05.

Compared to normoxic saline controls, α-SMA staining in the pulmonary vasculature was visibly higher in SuHx saline rats, indicating increased muscularisation in the disease model (Figure 4A). Visually, the higher levels of α-SMA staining in SuHx saline treated rats were reduced when rats were treated with macitentan or MM07. Subsequent quantification confirmed that, as expected, SuHx rats in the presence of saline showed significantly higher percentages of partially or fully muscularised vessels present in the lung versus normoxic/saline controls (Figure 4B). Importantly, treatment with MM07 significantly reversed the percentage of fully muscularised vessels to a level comparable to that observed in the normoxic/saline controls. Surprisingly, whilst there was a trend for macitentan to reverse vascular muscularisation, the percentages of partially or fully vascularised vessels did not differ significantly versus saline treated SuHx rats. Blood vessel smooth muscle scoring (Figure 4C) also confirmed that MM07, but not macitentan, significantly reduced vascular muscularisation.

Finally, tissue was assessed for the presence of neointimal lesions (Figure 4D), another characteristic symptom of PAH. A significantly higher number of lesions were observed in SuHx treated rats in the presence of saline versus normoxic/saline controls. Neither MM07, nor macitentan, significantly reversed the presence of lesions, although we did observe a trend towards a reduction in lesion number, particularly with MM07.

## 4 Discussion

Pathological hallmarks of PAH include increased vascular cell proliferation, migration, and remodelling of the extracellular matrix ((Taguchi and Hattori, 2013). Consequently, increases in mean arterial pressure, and downstream right ventricular hypertrophy, ultimately cause right ventricular failure (Thenappan et al., 2018; Ruopp and Cockrill, 2022). Current therapeutic strategies, such as those targeting prostanoid or nitric oxide pathways, aim to relieve pulmonary vasoconstriction and alleviate afterload on the right ventricle, but often do not reverse cardiovascular remodelling or provide direct benefits in the heart.

Stimulating the apelin pathway offers beneficial vasodilatory haemodynamic effects (Maguire et al., 2009), increases cardiac contractility (Perjes et al., 2014), has been shown to improve cardiac output in PAH patients (Brash et al., 2018), and is suggested to address underlying drivers of PAH disease progression (Falcao-Pires et al., 2009; Alastalo et al., 2011; Yang et al., 2017; 2019). However, a key limitation of apelin agonist therapy is β-arrestin dependent desensitisation of the target apelin receptor, necessitating the identification of G protein-biased ligands that offer better efficacy and cardioprotective effects (Brame et al., 2015; Read et al., 2016; 2021).

Here, we used the G protein-biased apelin receptor peptide ligand, MM07, and demonstrate reversal of key mechanisms and symptoms of PAH established using the validated SuHx model in rats. Crucially, the protective effects of MM07 were comparable, or even better, than the endothelin receptor antagonist, macitentan, which is a standard-of-care therapy used in PAH patients.

In this study, MM07 significantly reversed the increased Fulton index of SuHx rats (Figure 2), providing evidence that the drug protects against right ventricle hypertrophy, as characterised in PAH. Further, we also observed that MM07 significantly reduced right ventricular systolic pressure in SuHx rats (Figure 3), to an extent similar to macitentan.

MM07 reduced vascular remodelling in lung vessels (Figure 4). Interestingly, macitentan did not significantly reverse vessel muscularisation, pointing to MM07 potentially providing superior disease-modifying effects versus this standard-of-care drug in this animal model. Whilst drug treatments did not significantly reduce the number of neointimal lesions in lung tissue of SuHx rats, there was a trend particularly for MM07. Further studies may clarify whether apelin receptor therapy attenuates this morphological/pathological aspect of PAH. Overall, our data further support the potential for G protein-biased apelin receptor agonists as potential novel cardiovascular therapies.

The precise mechanisms underlying the disease-modifying effects of apelin receptor ligands are not fully understood, but anti-apoptotic and anti-proliferative actions have been observed in PAECs and PASMCs (Yang et al., 2019; Read et al., 2021), promoting vascular homeostasis that is perturbed in PAH (Alastalo et al., 2011; Frump et al., 2021). Additionally, apelin is anti-fibrotic in the heart, and directly suppresses expression of tumour necrosis factor-β and transforming growth factor-β mediated expression of myofibroblast markers (including α-smooth muscle actin and collagen) to attenuate myocardial fibrotic remodelling *in vivo* (Pchejetski et al., 2012; Sato et al., 2013). Intriguingly, apelin has been shown to prevent and alleviate crystalline silica-induced pulmonary fibrosis through inhibition of transforming growth factor-β (Shen et al., 2023), suggesting that apelin agonists might have synergistic effects when used with the transforming growth factor-β ligand trapping therapy, sotatercept, which is on track for FDA approval in PAH (Torbic and Tonelli, 2024).

Apelin receptor mediated vasodilatation is predominantly nitric oxide dependent in humans (Japp et al., 2008; 2010), suggesting treatment with apelin agonists could also work synergistically with sGC stimulators such as riociguat, or PDE5 inhibitors such as sildenafil and tadalafil, that are currently used to treat PAH patients. As proof-of-principle, a recent clinical study of apelin in PAH showed that administration of apelin provided significant additive benefit, on top of concomitant PDE5 inhibitor therapy, in decreasing pulmonary vascular resistance, and increasing cardiac output and stroke volume (Brash et al., 2018).

Notably, apelin agonists also work to physiologically antagonise endothelin mediated vasoconstriction (Maguire et al., 2009), which is exacerbated in PAH patients (Giaid et al., 1993). Whilst endothelin receptor antagonists such as macitentan are effective in reducing PAH morbidity and mortality (Pulido et al., 2013), the antagonist, bosentan, has been shown to decrease cardiac contractility in hypertrophied rat hearts (Nagendran et al., 2013), presenting a potential adverse effect in PAH. This suggests that the positive cardiac inotropic effects of apelin agonist treatment could be of use when employed as an adjuvant to macitentan, and further animal and clinical studies should aim to confirm this hypothesis.

In summary, our data demonstrate that the G protein-biased apelin receptor agonist, MM07, beneficially reduced RVSP and right ventricle hypertrophy in the SuHx rat model of PAH, which recapitulates features of the human disease. MM07 also significantly reversed pulmonary vessel muscularisation and showed a trend towards reducing the incidence of neointimal lesions. Therapeutic effects of MM07 were equivalent, or in some instances, superior to the standard-of-care endothelin receptor antagonist macitentan. Importantly, apelin agonists such as MM07 exert their effects on a pathway that is independent of those targeted by the current standard-of-care drugs, offering potential synergistic benefits whilst having a direct effect on cardiac function. The findings suggest that MM07 could be used to replace decreasing levels of endogenous apelin peptide observed in PAH, and could be employed as a potential adjuvant therapy in patients who do not respond sufficiently to standard-of-care treatment options.

## Data Availability

The raw data supporting the conclusion of this article will be made available by the authors, without undue reservation.
